# Caregivers of Neuromuscular Patients Living with Tracheostomy during COVID-19 Pandemic: Their Experience

**DOI:** 10.3390/jcm12020555

**Published:** 2023-01-10

**Authors:** Paola Pierucci, Eleonora Volpato, Francesca Grosso, Maria Luisa De Candia, Massimo Casparrini, Elena Compalati, Francesco Pagnini, Paolo Banfi, Giovanna Elisiana Carpagnano

**Affiliations:** 1 Cardiothoracic Department, Respiratory and Critical Care Unit Bari Policlinic University Hospital, 70121 Bari, Italy; 2 Section of Respiratory Diseases, Department of Basic Medical Science Neuroscience and Sense Organs, University of Bari ″Aldo Moro″, 70122 Bari, Italy; 3 Department of Psychology, Università Cattolica del Sacro Cuore—Milano, 20123 Milano, Italy; 4 IRCCS Fondazione Don Carlo Gnocchi, 20123 Milano, Italy

**Keywords:** caregiver, tracheostomy, COVID-19, neuromuscular disorders, home care, protective factors, caregivers’ burden

## Abstract

Background: During a pandemic, tracheostomy management in neuromuscular disease (NMD) patients can be complex. Methods: Using a multicentre, multiple case study approach, we sought perspectives through semi-structured interviews via hybrid quali-quantitative analysis. The qualitative analysis involved a semi-structured interview and the quantitative analysis the completion of a battery of questionnaires. Aim: To investigate the caregivers’ experiences, burden and beliefs regarding tracheostomy during the Italian COVID-19 pandemic. The following instruments were administered: Connor and Davidson Resilience Scale (CD-RISC-25); Acceptance and Action Questionnaire-II (AAQ-II); State-Trait Anxiety Inventory (STAI); Langer Mindfulness Scale (LMS); Zarit Burden Interview (ZBI). Results: Fifty-three caregivers (62.3% female, mean age 52.2 (SD = 18.2)) participated in the study. The more resilient the carers, the more they were psychologically flexible (r = 0.380, *p* = 0.014) and able to cope adaptively with the emergency (r = 0.378, *p* < 0.006). Similarly, perceived control was higher the more resilient they were (r = 0.897, *p* < 0.001). The main emotions emerging were isolation and loneliness (15; 34.88%). The perception of tracheostomy meant that it could be seen as a lifesaver or as a condemnation. Similarly, the relationship with health professionals moves from satisfaction to a feeling of abandonment over the course of the pandemic. Conclusions: These findings offer a unique opportunity to understand the point of view of caregivers of NMD patients living with tracheotomy during the COVID-19 pandemic, when going to the hospital may have been hampered.

## 1. Introduction

Throughout their disease, patients suffering from NMD and chronic respiratory insufficiency may encounter the possibility of undergoing tracheotomy and invasive mechanical ventilation (IMV) for acute related complications or worsened respiratory insufficiency [1,2]. While prolonged respiratory failure is probably the most common reason for performing tracheostomy, other indications such as decreased level of consciousness, poor airway protective reflexes, difficult management of secretions, requests of patients on 24 h noninvasive ventilation, severe alterations in physiology associated with trauma and medical illness are also indications for tracheostomy. With the development of less invasive tracheostomy techniques that can be performed safely at the patient’s bedside, the frequency of performing tracheostomy appears to be increasing. After a tracheostomy, major lifestyle adjustments are needed. Not only new medical issues, but tracheostomy also brings with it several negative social aspects. Tracheostomy indeed is one of the most traumatic surgeries because it affects the patient’s quality of life and because it addresses the basic needs of an individual such as breathing, communication, nutrition, sexuality, social, relationships and professional activity, but also body integrity, especially when surgery is perceived as a permanent disability [3]. The inability to communicate is one of the top stressors for patients with an artificial airway in critical care [4]. Loss of voice is associated with serious negative changes starting with mood, frustration, anger, stress, loneliness, isolation and vulnerability. Patients may feel mentally traumatized because they believe they are not able to convey a message and be understood [5]. Moreover, tracheostomy can seriously affect the patient’s body image. This is where real psychological challenges and dramas arise for the patient because self-image, sexuality, social relations and even psychological health are damaged [6]. Family caregivers have a significant role in the treatment of people with a tracheostomy, not only because they are an important part of managing patients with a tracheostomy tube, but also because they provide effective psychological and emotional support to patients undergoing treatment [7]. At the same time, it is often challenging for family caregivers [8], because they experience physical, psychological, social and financial impacts. Physical impacts experienced by family caregivers include tiredness, poor sleep quality and fatigue [9,10]; psychological impacts include anxiety and depression. These have been identified to be high in family caregivers [11], such that they may persist for up to a year and not decrease in intensity for some [12]. At the same time, family caregivers experience symptoms of strain and post-traumatic stress disorder [13] because the severity of the patient’s illness influences the magnitude of psychological symptoms experienced by themselves [10]. Stress, anxiety, strain and burden are often associated with caregivers. The emotional and physical health of caregivers can affect the well-being of patients with tracheostomy [6]. The COVID-19 pandemic situation has not contributed to an improvement in outlook, as the main changes in the perception of comfort due to COVID-19 have been concern about the quality of care received by healthcare staff and the impoverishment of interaction [14]. First and foremost, patients with tracheostomy are at a greater risk of contracting COVID-19 and they are more likely to require intensive treatment or to result in mortality once infected [15]. Besides the risk of being infected, the impact of COVID-19 exacerbated the sense of frailty, isolation and consequent deterioration in the quality of life of these patients and their caregivers. At a time when interventions and follow-up visits have been postponed due to the disruption of healthcare systems, this type of patient can be considered as a population at risk of significant distress [16,17]. This psychological burden associated with the direct and indirect effects of COVID-19 must not be overlooked because depression seems to be an important predictor of survival. A deterioration in both physical and emotional functioning during the lockdown can be considered clinically significant. This might be explained because government restrictions have forced the entire population to reduce their activities and to spend entire weeks being locked up at home. This harmed both psychological and physical states [18].

## 2. Objectives

The main aim of the present study was to explore the caregivers ‘experiences of people with NMD, e.g., Amyotrophic Lateral Sclerosis (ALS), Spinal Muscular Atrophy (SMA), Duchenne Muscular Dystrophy (DMD)], or tetra-paresis, concerning tracheostomy management during the COVID-19 health emergency in Italy. Specific objectives included: (1) investigating whether resilience, flexibility and dispositional mindfulness were correlated with lower anxiety, stress and burden scores; (2) exploring the emotional experiences and management of the caregivers; (3) investigating whether a finding related to caregivers’ emotional coping and experience, characterized by a higher number of positive terms and metaphors, is correlated with levels of resilience, flexibility, state anxiety and dispositional mindfulness.

## 3. Methods

### Ethics

This study received ethical approval from the Ethics Committee of the IRCCS Fondazione Don Carlo Gnocchi section of the IRCCS Regione Lombardia (reference: 8 September 2021) and the Ethics Committee of the Bari Policlinico University hospital (reference number 6747 of the 24 May 2021. Before the interview, participants provided their signed informed consent by mail. Oral consent was obtained also on the day of the interview.

## 4. Study Design and Methodological Orientation

A multicenter case series quanti-qualitative study was carried out according to the Consolidated Criteria for Reporting Qualitative Research Checklist (COREQ) [19].

The Interpretative Phenomenological Analysis (IPA) [20,21] was adopted as a paradigm to conduct the study. IPA focuses the attention on understanding the complex system of meanings related to a unique and subjective intimate phenomenon [22]. This inner phenomenon is characterized by the presence of beliefs and constructs made manifest or suggested by the interviewee’s discourse and may represent a piece of his or her identity. In both cases, meaning is central and the aim is to try to understand the content and complexity of these meanings rather than to measure their frequency.

## 5. Participants

### 5.1. Sampling

Participants were purposively sampled and recruited at the Heart and Respiratory Rehabilitation Unit of the IRCCS Fondazione Don Carlo Gnocchi of Milan, Centro Santa Maria Nascente and at the Cardio-Thoracic Unit of Policlinico of Bari. Each potential participant in the study was contacted by one of the researchers involved in the study by telephone call to present the purpose of the research and thus verify his/her availability. Then, at the time of the telephone call, arrangements were made to schedule a functional time and day for the caregiver for the next phase. The recruitment of participants proceeded until theoretical saturation of the sample, i.e., until a redundancy of themes was observed and no new themes emerged [23].

### 5.2. Inclusion and Exclusion Criteria

To take part in the study, participants had to be caregivers of people with a tracheostomy and a diagnosis of NMD such as ALS, SMA, DMD, or tetra-paresis. The requirements also included being a caregiver for at least two years (i.e., since the start of the COVID-19 pandemic) and being aware of the diagnosis and medical record of the family member for whom one is a caregiver. To avoid potential research bias, younger subjects (<18 years old) and those with recorded psychiatric disorders or cognitive impairment were excluded from the study.

### 5.3. Setting and Time

Data collection took place from January 2022 until March 2022. Questionnaires and interviews were carried out online or by telephone, because of the resurgence of infections at the time due to the pandemic, the distance of the researchers from the participants, and to accommodate the needs of the caregiver. All telephone interviews took place in a quiet room to ensure privacy and confidentiality.

### 5.4. Materials and Data Collection

The caregiver accessed via an online link, realized thanks to the use of Qualtrics’ platform, to a series of questionnaires designed to investigate the variables of interest. If the caregiver was unable to fill them out directly, he/she was assisted by a qualified and trained psychologist in completing them. The questionnaires took on average a maximum of 30 min to complete.

The following data were collected:

- Socio-demographic and clinical data: gender, level of education, profession (current or previous), marital status, role, how long they have been caring for their loved one and for how many hours/weeks, drug therapy taken, pathologies and/or comorbidities (if any).
- Psychological tests:
  (a) Connor and Davidson’s Resilience Scale (CD-RISC-25) [24], designed to detect resilience. The CD-RISC consists of five factors: 1. personal competence and tenacity (8 items); 2. self-confidence and management of negative emotions (7 items); 3. positive acceptance of change and secure relationships (5 items); 4. control (3 items); 5. spiritual influences (2 items). The Connor Davidson-Resilience Scale is based on a 5-point Likert scale, ranging from 1 “totally false” to 5 “totally true”. The Resilience Scale has good internal consistency with values of Cronbach’s alpha varying across research from a minimum of 0.82 to a maximum of 0.93. Its stability was also measured using the retest method at 24 weeks with equally positive results.
  (b) Acceptance and Action Questionnaire-II (AAQ-II) [25], designed to detect flexibility. The AAQ-II was developed to establish an internally consistent measure of the mental health and behavioral effectiveness model of ACT. The AAQ-II began as a 10-item scale, but after the final psychometric analysis it was reduced to a 7-item scale (2011). It was designed to assess the same construct as the AAQ-I and the two scales are correlated at 0.97, but the AAQ-II has better psychometric consistency.
  (c) State-Trait Anxiety Inventory (STAI) [26], designed to detect trait anxiety. The State-Trait Anxiety Inventory (STAI) is a psychological questionnaire based on a 4-point Likert scale and consists of 40 questions on a self-report basis. The ST,AI measures two types of anxiety—state anxiety, or anxiety about an event and trait anxiety, or level of anxiety as a personal characteristic. Higher scores are positively correlated with higher levels of anxiety. Its most recent revision is Form Y and is offered in 40 languages. The internal consistency coefficients for the scale ranged from 0.86 to 0.95; test–retest reliability coefficients ranged from 0.65 to 0.75 over a range of 2 months. The test–retest coefficients for this measure in the present study ranged from 0.69 to 0.89. This offers considerable evidence of the scale’s construct and concurrent validity.
  (d) Zarit Burden Interview (ZBI) [27], designed to detect caregiver burden. The Zarit Burden Interview (ZBI) is a 22-question questionnaire designed to measure the extent to which a caregiver perceives his or her level of burden because of caring for a person with a particular diagnosis. Initially developed to measure the stress associated with caring for elderly people living in the community, it has since been validated in many patient populations and is a common measure of caregiver burden. Based on the original 29-item scale, the ZBI has undergone several modifications that have led to the current 22-item assessment. The ZBI questions comprise 5 domains: (1) burden in the relationship (6 items), (2) emotional well-being (7 items), (3) social and family life (4 items), (4) finances (1 item) and (5) loss of control over one’s life (4 items). Most items explore both personal stress (12 items) and role stress (6 items). The ZBI uses a 4-point ordinal scale describing the degree of load experienced from 0 = never to 4 = almost always and takes about 10 min to complete. The maximum score is 88 with higher scores indicating a greater load.
  (e) Langer Mindfulness Scale (LMS) [28], to measure dispositional mindfulness. This is a questionnaire with 21 questions to be used as a training, self-discovery and research tool. It assesses four domains associated with mindfulness thinking: novelty seeking, engagement, novelty production and flexibility. An individual who seeks novelty perceives every situation as an opportunity to learn something new. An individual who scores high in engagement is likely to notice more details about his or her specific relationship with the environment. An individual who produces novelty generates new information to learn more about the current situation. Flexible people welcome a changing environment rather than resist it. The LMS has proven to have good test–retest reliability, factor validity and construct validity.

Furthermore, at the end of the previous phase, the participant underwent an ad hoc semi-structured interview (Table 1) aimed at investigating the experience of their loved ones’ tracheotomy itself and the period before and after it happened. Moreover, the management of the tracheostomy, and the burden and emotional experiences over the COVID-19 period were also explored. The researcher used recognized qualitative techniques, such as returning and facilitating dialogue and useful memos were collected to account for that contextual information (such as relationships, pauses and/or interruptions in speech). This interview, subject to consent and whether the participant showed signs of fatigue, was conducted in a second meeting without taking more than 30 min.

It should also be noted that both the above-mentioned tests and the semi-structured interview were administered with the support of a researcher by telephone if the participant did not have digital means or had difficulties using them.

### 5.5. Data Management

All interviews were audio-recorded, unwound and transcribed ad verbatim. The verbatim transcription took place in parallel with the collection of the interviews. To ensure anonymity, both questionnaires and interviews were pseudonymized with a subject-generated identification code (i.e., removing identifiable data to ensure privacy and confidentiality). Thus, no names were used during the later stages of the study. In particular, the uncoiling of the interviews was carried out respecting the criteria for the protection of anonymity, i.e., removal of first and last names, removal of names of places and people mentioned and removal of any details that might have made the study participants recognizable. In addition, the unwinding of all interviews took place under the supervision of the project doctor and was only carried out by the research team members. The interviews were then collected in a password-protected online file to which only the research team had access.

### 5.6. Data Analysis

Quantitative analyses were set up as statistical-descriptive to describe the sample. Based on the distribution of the variables of interest, parametric (or non-parametric) correlations were also carried out to identify the relationships between the experiences expressed in the semi-structured interviews, the specific characteristics of the sample and what emerged from the tests used. These analyses were conducted using Jamovi software (version 2.2.3).

Qualitative analyses were conducted on the semi-structured interviews, which will be audio-recorded, transcribed verbatim and anonymized, based on IPA, to identify themes, sub-themes, frequency of words used and metaphors. During the data analysis, which is flexible and dynamic, the IPA developers claim that researchers return to the interview data when necessary and that they focus on meanings during the analysis process. The main steps of analysis through IPA can be summarised as follows: reading and re-reading the text in depth; noting free associations and exploring semantic content; identifying themes and connections between them; identifying recurring sub-themes and their connections; noting idiosyncratic aspects of the interviews; noting metaphors and linguistic and temporal references. These analysis phases were followed by two of the group’s independent researchers. To address issues of reliability and credibility, the emerging themes were constantly compared and contrasted during regular meetings and, in the event of disagreements, a third researcher was involved. The final categorization of themes and definitions was agreed upon by the entire research team and data saturation and triangulation of researchers were achieved [29]. These analyses were conducted using NVivo software (version 12, QSR international^®^).

## 6. Results

### 6.1. Participant Demographics

**Figure 1:**
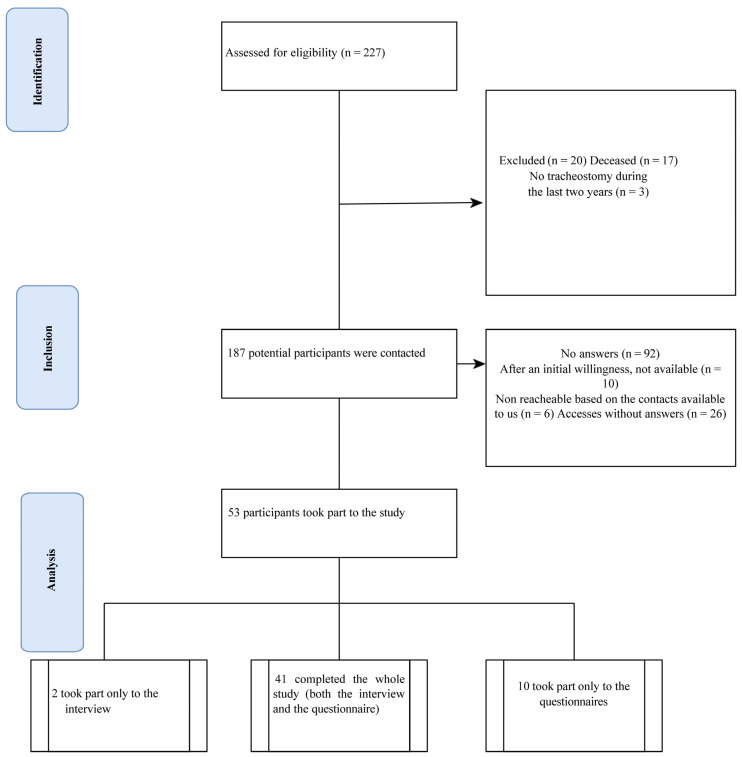
Prisma flow diagram of study participants [30].

The flow chart shows the process of recruitment and inclusion of the 53 participants (Figure 1). Their socio-demographic features are shown in Table 2.

In the caregivers who completed the questionnaires, trait anxiety and state anxiety were positively correlated (r = 0.397; *p* = 0.011). However, where psychological flexibility was greater, state (r = −0.509; *p* < *0*.001) and trait (r = −0.325; *p* = 0.038) anxiety levels tended to decrease significantly. Furthermore, the greater the levels of psychological flexibility, the greater the resilience (r = 0.378; *p* = 0.006) and, in particular, the sense of personal competence, tenacity (r = 0.447; *p* = 0.001), perception of control (r = 0.360; *p* = 0.009), trust in one’s instincts and tolerance of negative affects (r = 0.344; *p* = 0.013).

With an increase in dispositional mindfulness, there is also an increase in resilience (r = 0.380; *p* = 0.014). This finding is also useful in the face of the increase in resilience as the components of novelty producing (r = 0.502; *p* < 0.001) and novelty seeking (r = 0.427; *p* = 0.005) increase. In this regard, it is also worth noting that the increased presence of dispositional mindfulness is matched by an increase in tenacity (r = 0.343; *p* = 0.028) and self-confidence as well as tolerance of negative affect (r = 0.420; *p* = 0.006). In addition, as scores on the novelty-seeking component increased, so did tenacity (r = 0.345; *p* = 0.027) as well as confidence in one’s own instincts and tolerance of negative affect (r = 0.477; *p* = 0.002) and readiness to accept change (r = 0.329; *p*= 0.036). The more novelty production increases, the more there is an increase in tenacity (r = 0.427; *p* = 0.005), confidence in one’s instincts (r = 0.489; *p* = 0.001), ability to adapt to change (r = 0.312; *p* = 0.047) and control (r = 0.051; *p* = 0.003). Moreover, the greater the engagement, the greater the dispositional mindfulness (r = 0.556; *p* < 0.001) and psychological flexibility (r = 0.410; *p* = 0.008).

Paradoxically, where the caregiver’s overall burden is greater, resilience is also greater (r = 0.585; *p* < 0.001). This also tends to correlate positively with the components of tenacity (r = 0.609; *p* < 0.001), trust in one’s own instincts (r = 0.572; *p* < 0.001), control (r = 0.574; *p* < 0.001), adaptation to change (r = 0.481; *p* < 0.001) and spiritual influences (r = 0.359; *p* = 0.009). Instead, as psychological flexibility increases, the caregiver’s burden decreases (r = 0.648; *p* = 0.001). Perceived emotional well-being tended to be higher in the presence of greater resilience (r = 0.341; *p* = 0.013), in particular, toughness (r = 0.386; *p* = 0.005), trust in one’s instincts and tolerance of negative affect (r = 0.330; *p* = 0.017) and sense of control (r = 0.290; *p* = 0.037).

### 6.2. The Tracheostomy Experience through the Caregivers’ Eye

The experience of the tracheotomy changed in many ways the life of both patients and caregivers. In Table 3, the most salient information gathered through the interview related to the tracheotomy itself and the period immediately before and after is grouped. As can be seen, many caregivers report an experience of a tracheostomy performed in an emergency, unexpectedly (13; 24.52%). The majority expected an improvement in the quality of life for their loved one following the operation (9; 16.98%), thinking that their loved one would be able to return to managing at least some of the activities of daily life (e.g., carrying out some office duties; going to a restaurant) and that it would slow down the evolution of the pathology. For five people, the interviews also reveal the need for a tracheostomy as a watershed between life and death. Following the tracheostomy of one’s loved one, the panorama of experiences is multifaceted. Nine people (16.98%) emphasize an improvement in their quality of life, followed by eight (15.09%) who complain of being unable to communicate or to have lunch or dinner with their loved ones (8; 15.09%).

**Figure 2:**
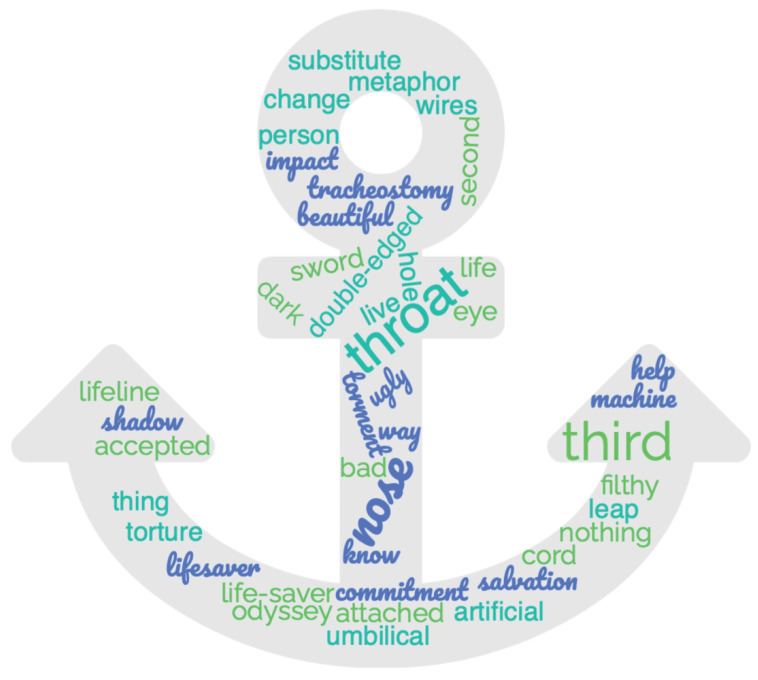
Tracheostomy metaphors.

Twenty-three (53.48%; 25 references) caregivers referred to tracheostomy in positive terms: among them, the most stressed value is its power to save a life. On the other hand, 13 caregivers (30.23%; 15 references) describe it in negative terms, such as a great condemnation or suffering. The majority of caregivers referred to the tracheotomy of their beloved ones as to a saving anchor; therefore the most frequently words and metaphors used by the caregivers for describing tracheostomy are grouped in Figure 2, featuring an anchor.

### 6.3. Caregivers Confronted with Tracheostomy at the Time of the Pandemic: Semi-Structured Interviews

As reported earlier, data from 43 semi-structured interviews were analyzed. Each interview lasted between 5 and 20 min, regardless of the context in which it was conducted (online or by phone). In Table 4 are shown the superordinate themes, themes and sub-themes that emerged from the analysis conducted. In the supplement (Table S1) the full table with examples of quotation is available.

### 6.4. Perceived Changes

One of the superordinate themes that emerged is inherent in the changes in health care received, which can be distinguished between those experienced during the period of lockdowns and related to the health emergency following them. In this regard, some found important differences, either precisely because of the absence of health personnel who could go to the home or because of the drastic decrease in contact with the home due to the lockdown (18 references; 324.29% coverage). For some, however, the differences were minimal because they covered short time brackets (e.g., of a few weeks) (16 references; 154.59% coverage) and, for others, no significant changes were seen because they did not have home care already in the pre-pandemic period (33 references; 301.8% coverage).

### 6.5. Coping Strategies

Another superordinate theme concerned coping strategies. Of these, the most widely used were those based on emotions (14; 32.55%; 26 references; 188.07% coverage), among which fear, anxiety and worry emerge most prominently, which either paralyze or cause caregivers to be activated in search of avoidance solutions to the dangers associated with contagion. This was followed by problem-based strategies (9; 20.93%; 16 references; 86.11% coverage) and, albeit to a lesser extent, those related to social support (5; 11.62%; seven references; 61.47% coverage) and passive adaptation (2; 4.65%; three references; 14.35% coverage; 61.47% coverage) (Table 4).

### 6.6. Emotions

The main emotion that emerged from the interviews was a sense of isolation and loneliness (15; 34.88%) together with a sense of abandonment (19; 44.18%). Significant, in particular, was the emotion of fear, which was connoted in three distinct modes: fear for the health of one’s loved one (10; 23.25%; 13 references; 72.29% coverage); fear of the unknown (2; 4.65%; two references; 5.95% coverage); and fear related to virus infection (16; 37.20%; 20 references; 94.95% coverage). Other emotions were referred to as worry, general anxiety, anger, distress and anxiety specifically related to the mass media’s information (Table 4).

### 6.7. Relationships

The superordinate theme for relationships (Table 4) was characterized by the presence of references to the absence of support (9; 20.93%; 12 references; 82.44% coverage), thus recalling the previously mentioned themes, such as the sense of protection and anxiety perceived by health care personnel (23; 53.48%; 37 references; 186.97% coverage), especially during the lockdown period.

### 6.8. Satisfaction

Few references are made to satisfaction concerning the care services received during the period of emergency health care. Only in a few cases are they described as very poor (1; 2.32%; 1 reference; 5.37% coverage) or as having improved (1; 2.32%; 1 reference; 1.94% coverage), while in most references caregivers stated that they felt no change (3; 6.97%; three references; 6.23% coverage) (Table 4).

### 6.9. Tracheo’s Changes

Few emotions (2; 4.65%; three references; 17.42% coverage) related to the changes introduced by tracheostomy were reported, while many were the reported changes in life and daily management (15; 34.88%; 34 references; 420.21% coverage) (Table 4). Main emotions include the fear of making a mistake, not arriving on time and not understanding the needs of one’s loved one.

Finally, a higher number of positive terms and metaphors was correlated with higher levels of resilience, flexibility, state anxiety and dispositional mindfulness.

## 7. Discussion

This study focused on assessing the experience of caregivers of NMD patients living with tracheotomies at home during the long pandemic period. To the best of our knowledge, no other study has ever focused on this topic. The decision of these researchers to carry this study toward the end of the series of pandemic waves was to collect as much information related to this long period of isolation as possible. This specific group of carers is often described as part of the NMD “family illness” [31] and may suffer intensively because of very demanding and overwhelming activities. Several studies have highlighted that they often experience heavy care burdens and psychological distress, so they need to implement coping strategies to manage or lighten stressful situations [32,33,34,35]. Since there are very few studies on the topic and in particular of carers of patients with NMD living with tracheotomy, this study is unique. Indeed, this is a very demanding role with heavy care burden which can be worsened by social isolation and fear of COVID-19 infection. During COVID-19 early stage of the pandemic, a previous study focused on evaluating the QoL of both ALS patients and caregivers and showed that while from one side there was not a significant reduction of QoL, for both people interviewed, on the other side the caregiver burden significantly increased mainly because of the reduction of family help for primary caregivers. Furthermore, the authors highlighted the importance of wide social support in the management of this clinical condition [36]. Given that this study was carried out towards the end of the COVID-19 pandemic waves, we decided instead not to focus on QoL but to understand the coping strategies that carers put in place. Interestingly, we found that, when the caregiver’s overall burden is greater, resilience is also greater. Similarly, this tends to correlate positively with the components of tenacity, trust in one’s instincts, control, adaptation to change and spiritual influences. Indeed, in our population, as psychological flexibility increases, the caregiver’s burden decreases. Perceived emotional well-being tended to be higher in the presence of greater resilience, in particular, toughness, trust in one’s instincts, tolerance of negative affect and a sense of control. Therefore, the prevalence of carers increased forced autonomy due to the pandemic and isolation improved their feeling of resilience. This was perceived despite most of the interviewed caregivers describing that there was no change in the real day-by-day management, therefore it was mainly a subjective perspective change. It was making a virtue of necessity via which carers escaped the worse. In terms of coping strategies, the most widely used by carers were those based on emotions, among which fear, anxiety and worry emerge most prominently, which either paralyse or cause caregivers to be activated in search of avoidance solutions to the dangers associated with contagion. This was followed by problem-based strategies and, to a lesser extent, those related to social support and passive adaptation. Interestingly a study by Siciliano et al. [37] described that ALS caregivers who adopted the emotion-oriented coping strategy were those with higher levels of psychological distress compared to those who adopted task-oriented strategies. However, when specific psychological interventions are offered to ALS caregivers they seem not to have any effect on psychological distress, burden, quality of life and on patients’ psychological distress, although a significant positive effect was revealed on caregivers’ feelings of control over caregiving [38]. Interestingly, very few studies have addressed the topic of psychological intervention in this specific population of people, therefore there is a much-needed research gap to bridge. The results of these small studies showed that shared experiences allowed participants to feel less alone, better understood and, more accepting of their beloved ones [39]. Interestingly, there was a long time-frame between the NIV initiation and the tracheotomy (Table 2) which confirms the important value of NIV even for prolonged time during the day (more than 14 h); but also enhances the importance of a correct follow up (in person or in telemedicine) specifically in these patients [40,41]. The NIV success is related to the right choice of interface which may vary over the years of use and the strict follow up allow the right timing for transitioning to tracheotomy instead of an emergency procedure [42]. Indeed, the experience of the tracheotomy for a significant part of patients included in this study, almost 25%, was rushed and linked to an emergency procedure without much explanation of details. The perceived feeling of their caregivers was positive as it allowed the patient to survive, but on the other, the hand they describe a sudden change, worsening QoL for them and their loved ones. The reasons behind this sudden change may be found in the lack of usual respiratory follow-up during the pandemic period. Indeed, mouth patient facilities were not available as the majority of the respiratory healthcare providers were caring for the disproportioned number of sick patients with severe respiratory COVID-19 infection. Undoubtedly, the advent of telemedicine has lightened the burden and proved to be effective in CRF patients. However, most of the studies reported the lack of in-person visits as a disadvantage, probably due to the unique patient–doctor relationship that is encountered among this patient population. Globally, telemedicine helped patients and caregivers not to feel abandoned [39,43,44,45,46,47]. The COVID-19 outbreak prompted wider use of telemedicine services, suggesting that telemedicine for NMD patients and carers may be used as replacement when the emergency requires it, but otherwise may become complementary to in-person care. This is true except for the most vulnerable patients, including those with tracheotomy and HIMV and their caregivers. In these categories, telemedicine may be considered to replace the usual in-person care in the referring center, depending on circumstances and patient/career preferences, with the help of structured integrated care with a multidisciplinary team of specialists and general physicians and local/home-based health care providers [39,48]. It is also to be considered that a diverse relationship between caregiver and patient (spouse vs. parent of the patient) may vary the approach to the problem and the related outcome. Despite this was not the aim of the present study, it offers a relevant insight for future studies and considerations.

While considering the relevant findings of this study, some limitations may be noted. First of all, IPA allows us to delve into people’s subjective experiences, which would otherwise not be accessible. However, given the degree of depth, it requires a limited sample of people and the involvement of the researcher in the interpretation of experiences during data analysis. To minimize possible bias, as mentioned above, the analyses were conducted independently by two researchers and questionable topics were discussed with a third. Another limitation of the study is that it is descriptive and not interventionist. However, these results are necessary to create a snapshot of what happens when, in emergencies such as COVID-19, caregivers and their loved ones are objectively more isolated and have less opportunity to get out of the house and be welcomed in their needs. Structuring both longitudinal and randomized controlled trials would also help to improve healthcare management in high and urgent complex contexts. Finally, the small purposive sample limits the possibility of the generalizability of the results. However, given the rarity of the disease considered, a much larger population sample would be very difficult to achieve.

Continuous family interventions are needed, on a day-by-day basis about how to keep them informed, engaged and constantly supported by the different HCPs, even if in emergencies. Telehealth monitoring, as other studies have shown, could be relevant to sustain caregivers and should be incorporated into standard healthcare approaches.

## 8. Conclusions

This study purposed to give a snapshot of the role of the caregivers of NMD patients living with tracheotomy during the long pandemic period. The picture drawn is of people with a high burden of care which was worsened by the pandemic. Therefore, more support needs to be put in place to alleviate their stressors. Moreover, telemedicine needs to be implemented and focus not just on NMD and chronic respiratory failure patients but also on caregivers, and more studies should address effective intervention to lighten the burden of this “family illness” and its care.

## Figures and Tables

**Table 1:** Interview guide.

Semi-Structured Interview Question
1. What do you think has changed in the management of tracheotomy during COVID-19?
2. How is the home care received during the period of the medical emergency?
3. How do you feel/are you feeling about this management?
4. What kind of changes have you noticed in your relationship with home healthcare professionals?
5. What difficulties are you experiencing with the management of the tracheotomy during the health emergency?
*Possible prompts*: When?/How often?/Physical?/Emotional?/Practical?
6. What difficulties are you experiencing with the management of the tracheotomy during the lockdown period?
*Possible prompts*: When?/How often?/Physical?/Emotional?/Practical?
7. What emotions are you predominantly experiencing during the medical emergency period?
*Possible prompts*: Can you think of specific situations?
8. What emotions are you predominantly experiencing during the lockdown period?
*Possible prompts*: Can you think of specific situations?
9. Which metaphor would you used to describe tracheostomy?

**Table 2:** Socio-demographic information on the participants.

Variables	Levels	N (%)	M (SD)
**Total (N)**		53 (100%)	
**Age (M, SD)**			52.2 (18.2)
**Gender (n, %)**	Men	19 (35.8%)	
	Women	33 (62.3%)	
	Prefer not to say	1 (1.9%)	
**Marital Status (n, %)**	Married	39 (73.6%)	
	Divorced	2 (3.8%)	
	Separated	1 (1.9%)	
	Single	5 (9.4%)	
	Widower	2 (3.8%)	
	Other	4 (7.5%)	
**Education (n, %)**	Primary School	5 (9.6%)	
	Secondary School	9 (17.3%)	
	High School	23 (44.2%)	
	Bachelor’s degree	6 (11.5%)	
	Master’s degree	8 (15.4%)	
	Other Specialisations (e.g., PhD)	1 (1.9%)	
	None	1 (1.9%)	
**Kind of job practiced before the diagnoses of the loved ones**	Self-employed	7 (13.20%)	
	Housewife	6 (11.32%)	
	Teacher	3 (5.66%)	
	Engineer	3 (5.66%)	
	Doctor	2 (3.77%)	
	Business Consultant	2 (3.77%)	
	Employee	10 (18.86%)	
	Retired	2 (3.77%)	
	Other	18 (33.96%)	
**Age of the dear ones M, (SD)**			50.2 (21.2)
**Gender** **of the dear ones (n,%)**	Men	9 (40.9%)	
	Women	11 (50%)	
	Prefer not to say	2 (9.1%)	
**Disease of the dear ones**	Amyotrophic Lateral Sclerosis (ALS)	18 (33.9%)	
	Spinal Muscular Atrophy (SMA)	4 (7.54%)	
	Congenital Myopathies	1 (1.9%)	
	Multiple Sclerosis (MS)	2 (3.77%)	
	Duchenne Muscular Dystrophy (DMD)	1 (1.9%)	
	Muscular Dystrophy	1 (1.9%)	
	Encephalopathy	2 (3.77%)	
	Tetra-paresis	3 (5.66%)	
	Other	21 (39.62%)	
**Kind of onset (only in case of ALS)**	Bulbar	6 (33.3%)	
	Spinal (lower limbs)	8 (44.4%)	
	Spinal (upper limbs)	2 (11.1%)	
	Respiratory	2 (11.1%)	
**Non-Invasive Ventilation (NIV) before tracheostomy**	Yes	22 (41.5%)	
	No	14 (26.41%)	
	I don’t know	4 (7.54%)	
	No answer	13 (24.52%)	
**Where did you try NIV for the first time?**	At the hospital	19 (35.84%)	
	Outpatient clinic	2 (3.77%)	
	No answer	32 (60.37%)	
**Problems with NIV**	Conjunctivitis, connective or corneal ulcers	1 (1.88%)	
	Skin abrasions or ulcerations due to the mask	6 (11.32%)	
	Dry nose and mouth	2 (3.77%)	
	Decubitus	2 (3.77%)	
	Airways obstruction	2 (3.77%)	
	Other	7 (13.20%)	
	No answer	33 (62.3%)	
**Hours of NIV’s usage before tracheostomy**			15.1 (8.11)
**Years of disease**			13.8 (14.4)
**Years from diagnosis**			9.58 (11.5)
**Diagnosis-tracheostomy time (Days)**			4218 (7776)
**Percutaneous Endoscopic Gastrostomy (PEG)**	Yes	35 (66.03%)	
	No	9 (16.98%)	
	No answer	9 (16.98%)	
**Use of cough assistant**	Yes	5 (9.43%)	
	No	12 (22.64%)	
	I don’t know	2 (3.77%)	
	No answer	34 (64.15%)	
**Phonatory valve during open ventilation**	Yes	10 (18.86%)	
	No	30 (56.6%)	
	I’ve tried it in the past, but I can’t use it	3 (5.66%)	
	No answer	10 (18.86%)	
**Use O2 or not**	Yes	19 (35.84%)	
	No	27 (50.94%)	
	No answer	7 (13.2%)	
	How much?		0.93 (0.90)

Notes. M = Mean; SD = Standard Deviation; N = The relationship between resilience, flexibility and dispositional mindfulness with lower anxiety, stress and burden scores.

**Table 3:** Beliefs and information related to before and after a tracheotomy as perceived by the caregivers.

**The Tracheostomy to Your Loved One Was Done**	**N (%)**
After extensively discussing it with doctors…	9 (16.98%)
After having extensively discussed it with the Doctors and a Psychologist…	10 (18.86%)
In an emergency, but I knew it could happen	12 (22.64%)
In case of urgency, absolutely unexpected	13 (24.52%)
Other	2 (3.77%)
No answer	7 (13.20%)
**Before your loved one received the tracheostomy, you thought that…**	
His/Her quality of life would have improved	9 (16.98%)
He/She would be able to resume and/or continue my activities of daily living (e.g., at home, with my loved ones, work…)	6 (11.32%)
I feel that he/she has many more years ahead of him/her	6 (11.32%)
He/She can no longer communicate verbally	6 (11.32%)
He/She can no longer eat	6 (11.32%)
Other	2 (3.77%)
No answer	18 (33.96%)
**After your loved one received the tracheostomy, it happened that…**	
My quality of life has been improved	9 (16.98%)
I was able to resume and/or continue to carry out my activities of daily life (e.g., at work, at home, with my dear ones…)	5 (9.43%)
I feel he/she would have many more years ahead of him/her	9 (16.98%)
I cannot longer communicate with him/her	8 (15.09%)
I cannot longer have lunch/dinner with him/her	8 (15.09%)
Other	3 (5.66%)
No answer	11 (20.75%)

**Table 4:** Organisation of superordinate themes, themes and subthemes emerging from the analyses.

Superordinate Themes	Themes	Subthemes
Changes (43; 100%; 167 references)	Perceived changes in the assistance during the lockdowns (43; 100%; 141 references)	Big differences (18; 41.86%; 35 references)
		Medium differences (16; 37.20%; 21 references)
		No differences (33; 76.74%; 85 references)
	Perceived changes in the assistance after the lockdowns (19; 44.18%; 26 references)	Confusion (2; 4.65%; 3 references)
		Getting Better (4; 9.30%; 4 references)
		Persistence (5; 11.62%; 7 references)
		Restart (8; 18.6%; 12 references)
Coping Strategies (30; 69.76%; 52 references)	Emotion-focused (14; 32.55%; 26 references)	
	Passive adaptation (2; 4.65%; 3 references)	
	Problem focused (9; 20.93%; 16 references)	
	Social support (5; 11.62%; 7 references)	
Emotions (43; 100%; 79 references)	Caregivers’ emotions (43; 100%; 71 references)	Abandoned (19; 44.18%; 33 references)
		Anger (2; 4.65%; 2 references)
		Anxiety (8; 18.60%; 12 references)
		Distress (1; 2.32; 2 references)
		Fear (12; 27.90%; 21 references)
		Anxiety related to the mass media (1; 2.32%; 1 reference)
	Others’ emotions (6; 13.95%; 8 references)	Frightening (6; 13.95%; 8 references)
Relationships (32; 74.41%; 50 references)	Abandoned (Covid-19 or not) (9; 20.93%; 12 references)	
	With others, the Health Care Professionals (23; 53.48%; 37 references)	
Satisfaction (5; 11.62%; 5 references)	Bad (1; 2.32%; 1 reference)	
	Same as before (3; 6.97%; 3 references)	
	Getting better (1; 2.32%; 1 reference)	
Tracheo’s changes (17; 39.53%; 37 references)	Emotion related to tracheo’s changes (2; 4.65%; 3 references)	

## Data Availability

The datasets generated during and/or analyzed during the current study are available from the corresponding author upon reasonable request.

## Supplementary Materials

The following supporting information can be downloaded at: https://www.mdpi.com/article/10.3390/jcm12020555/s1, Table S1: Organisation of superordinate themes, themes and subthemes emerging from the analyses, together with examples of quotations.

## Abbreviations

ALS	Amyotrophic Lateral Sclerosis
SMA	Spinal Muscular Atrophy
DMD	Duchenne Muscular Dystrophy
COPD	Chronic Obstructive Pulmonary Disease
ICU	Intensive Care Unit
COREQ	Consolidated Criteria for Reporting Qualitative Research Checklist
IPA	Interpretative Phenomenological Analysis
CD-RISC-25	Connor and Davidson’s Resilience Scale
AAQ-II	Acceptance and Action Questionnaire-II
STAI	State-Trait Anxiety Inventory
ZBI	Zarit Burden Interview
LMS	Langer Mindfulness Scale
NMD	Neuro muscular Disease
