# Supplementary material for: Caregivers of Neuromuscular Patients Living with Tracheostomy during COVID-19 Pandemic: Their Experience
Source: J Clin Med. 2023 Jan 10;12(2):555. doi: 10.3390/jcm12020555 (PMC9862176; doi:10.3390/jcm12020555)
Supplement: Supplementary file 1 [file jcm-12-00555-s001.zip › jcm-2051547-supplementary.pdf]

Journal of
Clinical Medicine

MDPI

Supplement Table S1: Organisation of superordinate themes, themes and subthemes emerging from the analyses, together with examples of quotations.

**Superordinate Themes**	**Themes**	**Subthemes**	**Examples of citations**
Changes (43; 100%; 167 references)	Perceived changes in the assistance during the lockdowns (43; 100%; 141 references)	Big differences (18; 41.86%; 35 references)	*<<...has changed a lot, we used to do only the essentials and the bare essentials, we used to linger a little longer, instead, in those days it was all pretty quick…>>* (Caregiver 11)
		Medium differences (16; 37.20%; 21 references)	*<<...maybe there was a time when they couldn't come, but it was a brief moment, yes, always because of this fear of...of contagion…>>* (Caregiver 10)
		No differences (33; 76.74%; 85 references)	*<<Unfortunately, we do not have this service here, since March they have been coming to do the cannula and PEG change at home, but until now we have always done it in the hospital....with Covd-19 everything has remained as it was>>* (Caregiver 16)
	Perceived changes in the assistance after the lockdowns (19; 44.18%; 26 references)	Confusion (2; 4.65%; 3 references)	*<<...nursing care is what resumed much later, in hiccups, because of the absence of nursing staff. Then they also put in people like that, with no experience, so in the end we felt a little bit like guinea pigs in this situation, we suffered this situation...>>* (Caregiver 8)
		Getting Better (4; 9.30%; 4 references)	*<<Then it changed over time, when they started coming home again to take things into their own hands, now the physical therapy hours are five and no longer three>>* (Caregiver 12)
		Persistance (5; 11.62%; 7 references)	*<<No, no still now because we must never let our guard down. His pulmonologist then always told us to protect him, even before the Covid, and at every slightest symptom to wear masks but that even before because then we risk infecting him even with the slightest cold because he is very delicate as a subject>>* (Caregiver 20)
		Restart (8; 18.6%; 12 references)	*<<After the vaccines we took everything back, we are back to normal>>* (Caregiver 7)

Coping Strategies (30; 69.76%; 52 references)	Emotion-focused (14; 32.55%; 26 references)		*<<At first we were afraid, no one came to visit us, in fact, even before the lockdown we told people who came to visit my wife not to come at all>>* (Caregiver 16)
	Passive adaptation (2; 4.65%; 3 references)		*<<It was an adjustment to everything and then we were almost back to normal>>* (Caregiver 3)
	Problem focused (9; 20.93%; 16 references)		*<<I also bought something of my own, the shoe cover, however, it was more us who stopped physical therapy and the psychologist>>* (Caregiver 11)
	Social support (5; 11.62%; 7 references)		*<<We felt close, the doctors came alongside us, we did not feel alone but we felt supported, we were happy because at that moment we were not alone but we knew how to handle the situation because at the beginning we were very upset, we were afraid something might happen however thanks to the support they gave us we made it>>* (Caregiver 20)
Emotions (43; 100%; 79 references)	Caregivers' emotions (43; 100%; 71 references)	Abandoned (19; 44.18%; 33 references)	*<<Also because there was an operator who had become positive, so we were completely abandoned without any staff>>* (Caregiver 1)
		Anger (2; 4.65%; 2 references)	*<<No, no even now, I for two years I never got any assistance at home, it's crazy, I would feel like calling the carabinieri, I know...at first it was not easy for me, because I didn't make the transition to the ward, I didn't have him for more than two hours with me and then they took him directly home with the ambulance and, so, I was in a lot of trouble at first, I didn't know where to put my hands either, then slowly you get used to it. If I had to have them now at home, I wouldn't even want them as gifts. By the way they used to send me an assistant who didn't know how to handle the tracheo, couldn't handle the tube and whatnot....>>* (Caregiver 26)
		Anxiety (8; 18.60%; 12 references)	*<<Clearly to summarize everything in one word is very complicated, there was a lot of fear and a lot of worry, a lot>>* (Caregiver 4)
		Distress (1; 2.32; 2 references)	*<<Stressati è il termine migliore, sempre sul chi va là>>* (Caregiver 42)
		Fear (12; 27.90%; 21 references)	*<<Fear, depression>>* (Caregiver 39)

		Anxiety related to the mass media (1; 2.32%; 1 reference)	*<<The anxiety that accompanied us in the first lockdown is also a little bit everything we were seeing on TV, the news loaded us with anxiety, worry even, in thinking about how we were going to solve important situations, like cannula change, PEG change, those things like that>>* (Caregiver 11)
	Others' emotions (6; 13.95%; 8 references)	Frightening (6; 13.95%; 8 references)	*<<…was kind of mishandled because everyone was scared…>>* (Caregiver 1)
Relationships (32; 74.41%; 50 references)	Abandoned (Covid-19 or not) (9; 20.93%; 12 references)		*<<What can we say, though? Many doctors, unfortunately, ignore most of these rare diseases, and I tell them with much pain, it is certain, however, that health care is not in good hands>>* (Caregiver 12)
	With others, the Health Care Professionals (23; 53.48%; 37 references)		*<<More attention, they all used to come with...the mask, with the mask they still come now, but the apron, maybe even now, plus the physical therapist and whoever goes close. Since the epidemic they are more afraid to infect, they have a lot of regard>>* (Caregiver 14)
Satisfaction (5; 11.62%; 5 references)	Bad (1; 2.32%; 1 reference)		*<<In the first period it was kind of mishandled because everyone was scared so, let's say, in short>>* (Caregiver 1)
	Same as before (3; 6.97%; 3 references)		*<<No problem whatsoever, absolutely. In fact, I am really very satisfied with the work done in all these three years including the period when this pandemic came up>>* (Caregiver 4)
	Getting better (1; 2.32%; 1 reference)		*<<Then it slowly got better>>* (Caregiver 1)
Tracheo's changes (17; 39.53%; 37 references)	Emotion related to tracheo's changes (2; 4.65%; 3 references)		*<<The fact of being afraid of making a mistake, of not remembering the things that were said to us, even though we actually wrote everything down. The fact that you take over this emotional part, so taking over the emotional part maybe, just maybe, you go a little bit anxious because you say 'oh my God, what if now I can't do this and do that other', then you stop and rationalize and you manage to do everything. Now it's like it's all acquired, from December 28 that we came home to now, in quotes we are much more confident, me and also the guy who helps us at home. So we do everything much more automatically, we*

			don't have that fear that we had in the beginning>> (Caregiver 17)
